# Prevalence of anxiety, depression, and post-traumatic stress disorder among paramedic students: a systematic review and meta-analysis

**DOI:** 10.1007/s00127-024-02755-6

**Published:** 2024-09-12

**Authors:** Adnan Alzahrani, Chris Keyworth, Khalid Mufleh Alshahrani, Rayan Alkhelaifi, Judith Johnson

**Affiliations:** 1 School of Psychology, University of Leeds, Leeds, LS29JT UK; 2 Department of Basic Science, Prince Sultan bin Abdulaziz College for Emergency Medical Services, King Saud University, 11466 Riyadh, Saudi Arabia; 3 Faculty of Arts and Humanity, Psychology Department, King Abdulaziz University, Jeddah, Saudi Arabia; 4 Department of Aviation and Marines, Prince Sultan bin Abdulaziz College for Emergency Medical Services, King Saud University, 11466 Riyadh, Saudi Arabia; 5 Bradford Institute for Health Research, Bradford Royal Infirmary, Bradford, UK; 6 School of Public Health and Community Medicine, University of New South Wales, Sydney, Australia

**Keywords:** Paramedicine, Paramedicine student, Paramedic, Common mental disorder, Meta-analysis, Prevalence

## Abstract

**Purpose:**

There are elevated mental health concerns in paramedic students, but estimates vary between studies and countries, and no review has established the overall prevalence. This systematic review addressed this by estimating the global prevalence of common mental health disorders, namely anxiety, depression, and post-traumatic stress disorder (PTSD), in paramedic students internationally.

**Methods:**

A systematic search of six databases, including MEDLINE, EMBASE, PsycINFO, CINAHL, Scopus, and medRxiv, was conducted to identify studies relating to mental health among paramedicine students. The search encompassed studies from inception until February 2023. To be considered for inclusion in the review, the studies had to report prevalence data on at least one symptom of anxiety, depression, or PTSD in paramedicine students, using quantitative validated scales. The quality of the studies was assessed using Joanna Briggs Institute (JBI) Checklist, which is a specific methodological tool for assessing prevalence studies. Subgroup analyses were not conducted due to insufficient data.

**Results:**

1638 articles were identified from the searches, and 193 full texts were screened, resulting in 13 papers for the systematic review and meta-analysis. The total number of participants was 1064 from 10 countries. The pooled prevalence of moderate PTSD was 17.9% (95% CI 14.8–21.6%), anxiety was 56.4% (95% CI 35,9–75%), and depression was at 34.7% (95% CI 23.4–48.1%).

**Conclusion:**

This systematic review and meta-analysis has found that paramedicine students globally exhibit a high prevalence of moderate PTSD, anxiety, and depression. The prevalence of these mental health conditions surpasses those among paramedic providers and the general population, as indicated by previous reviews. Further research is therefore warranted to determine appropriate support and interventions for this group.

**Supplementary Information:**

The online version contains supplementary material available at 10.1007/s00127-024-02755-6.

## Introduction

Mental health disorders are common in the general population, with anxiety affecting around 301 million (4% of the global population) according to the Global Burden of Disease Study in 2019 [18]. Similarly, depression affecting around 280 million [52]. The underlying causes of mental health disorders are complex, but evidence suggests that risk of these is elevated in occupational groups who experience potentially stressful events in the course of their work [57, 59, 57]. One such high-risk group is paramedics [20]. Several studies have found a higher rate of mental health issues (e.g., depression, PTSD, stress) among paramedics than in the general population [4, 5, 29, 36, 47]. For example, in Saudi Arabia, the prevalence of anxiety and depression among the general population is between 12.4% and 12.7% respectively [1, 2]. However, for paramedics and paramedic students, the rates of anxiety are 19.3% [3] and 24.3% [6].

Paramedics are a fundamental part of the healthcare system, enacting clinical and non-clinical roles in a variety of unscheduled and dynamic settings (e.g., prehospital; [13, 54]). Thus, they face innumerable challenges as they must handle different cases in unpredictable contexts [10, 26] and be ready to make life-or-death decisions in a limited time frame. Some of the greatest burdens to paramedics’ mental health are found in their daily tasks and work environments, including attending to traumatic cases (e.g., death, severe trauma), lack of resources, and long shifts [4, 5], 58, 20] [33]. These burdens pose significant challenges, including related physical and mental demands, and so it is unsurprising that the risk of common mental health disorders is elevated in this group [4, 5, 24, 40].

Internationally, the training for paramedics involves practical clinical placements [14]. As such, paramedic students also face similar challenges to qualified paramedics [16]. Furthermore, paramedic students encounter additional challenges related to academic requirements and training placements [7, 16, 26]. A limited number of studies have examined the mental wellbeing of paramedic students and their findings have suggested that mental health disorders are more prevalent among paramedic students than the general population [6, 16, 36]. However, the global prevalence of mental health problems among paramedic students is unclear. Compared to pharmacy, medicine, and nursing students [27, 46, 48], respectively), paramedicine is an understudied major health speciality in tertiary education, with no current systematic review investigating the prevalence of mental health issues among the paramedic student population [19]. Quantifying the global rate of mental health disorders among paramedic students could highlight the extent to which addressing this should be prioritised by policymakers, researchers, clinicians, and tertiary academicians to understand the mental health needs of paramedic students [19].

Accordingly, this systematic review aimed to estimate the prevalence of common mental health disorders (i.e., anxiety, depression, PTSD) among paramedic students internationally [30, 53].

## Methods

The current systematic review followed the PRISMA statement and the Institute of Medicine’s Standards for Systematic Reviews [42, 34]. A protocol was registered in the PROSPERO International Register of Systematic Reviews (Registration No: CRD42022303570). To identify relevant studies of mental health issues among paramedic students and associated variables, major health databases were searched from inception to January 30, 2022, with the search updated on February 12, 2023.

### Search strategy

To identify relevant citations for inclusion, six databases were searched: CINAHL (EBSCOhost), EMBASE (ELSEVIER), Medline (Ovid), PsycINFO, Scopus, medRxiv (grey literature and pre-prints from bioRxiv and medRxiv). To identify relevant studies with data on mental health disorders among paramedic students and associated variables, the databases were searched from inception to January 30, 2022, with the search updated on February 12, 2023. The search strategy included Medical Subject Headings (MeSH) terms and keywords/phrases describing the population and the outcome; a language restriction was also placed on potentially relevant records. Further, the researchers manually searched reference lists and citation chaining of the included articles, which helped identify additional relevant articles. The search results from each database were exported, and the duplicates were removed. Only articles in the English language were included. All searches employed two main search blocks: mental health (anxiety, depression, and PTSD) and paramedic students.

### Eligibility criteria

The criteria for studies to be eligible for inclusion regarding population were if they only included participants enrolled in a paramedicine academic training programme and if they excluded qualified paramedics and volunteers. No interventions or comparators were applied in the review, and all quantitative study designs were included. Where studies reported more than one measurement of the outcome variables of interest (i.e., in the case of cohort/intervention studies), we included baseline measurements in our analysis. Additionally, mixed-method studies with quantitative data were also considered. The inclusion criteria included studies that measured anxiety, depression, or PTSD symptoms using any type of quantitative design, including grey literature. The exclusion criteria included qualitative studies without any quantitative element and studies that did not use validated questionnaires to measure outcome variables. The review’s primary outcomes were the prevalence of anxiety, depression, and PTSD among paramedic students, and there were no secondary outcomes.

### Study selection

The search results from each database were exported to Endnote X8.2 (Clarivate Analytics, Philadelphia, United States), and all duplicates were removed. The study selections were completed in two stages: in the first stage, the titles and abstracts of the identified studies were screened; in the second stage, the full texts of the retained studies were accessed and further screened according to the eligibility criteria. A percentage of titles/abstracts (10%) was screened independently by two reviewers to check for agreement (KA and RA). To estimate the level of agreement, we calculated the Kappa score, which indicated good agreement (k = 0.739). The remaining screening of titles/abstracts against the selection criteria was undertaken by two reviewers (AA and RA). Three independent reviewers each undertook the full-text screening (AA, KA, and RA). All disagreements were resolved through discussion; there was 100% agreement between reviewers on the second review.

### Data extraction

A data extraction form was devised in Excel 2016 16.7 (Microsoft Inc.) and piloted with five randomly selected studies. The quantitative data for the meta-analysis were extracted in a separate Excel file. The following descriptive information was extracted from the eligible studies: (1) study (country, recruitment methods, research design), (2) participants (age, gender, sample size, setting), (3) outcome variables (assessments to measure anxiety, depression, and/or PTSD and the reported prevalence of each outcome). The data were extracted by AA and reviewed by CK, JJ, KA, and RA.

### Quality assessment

The quality of the studies was assessed independently by two reviewers using the Joanna Briggs Institute (JBI) Critical Appraisal Checklist for Studies Reporting Prevalence Data [60]. Through this instrument, study quality was assessed across nine domains: the suitability of the sample to represent the target population, the recruitment methods, the sample size, the identification of the sample and the subjects, the data analysis approach used for the sample, the methods chosen to identify the outcome, the measurement of the condition, the appropriateness of the statistical findings, and the adequacy of the response rate. The nine items can be answered with ‘Yes’, ‘No’, ‘Unclear’, or ‘Not Applicable’. All disagreements between authors were resolved through discussion.

### Data analysis

All studies included in this project are described in a narrative review, including a table that quantifies our primary outcomes, design, and participant characteristics (see Table 1). The methods of the included studies varied, with different scales and cut-off scores implemented to identify the prevalence of anxiety, depression, and PTSD. To ensure an accurate interpretation, the prevalence of the selected mental health disorders was estimated using the moderate and above cut-off levels recommended on each of the scales.

**Table 1:** Characteristics of studies, and population included in the review

Study characteristics	Number of studies a (n/%)
Year of publication (n = 13)	1995–2005 (2/15%)
	2006–2015 (1/8%)
	2016–2019 (4/31%)
	2020- 2023 (6/46%)
Continent (n = 13)	Asia (3/23%)
	Africa (2/ 15%)
	North America (2/15%)
	Europe (1/ 8%)
	Oceania (5/39%)
Sample type (n = 13)	Only paramedicine students (7/54%)
	Paramedic students among other university students (3/23%)
	Paramedic students among paramedic providers (3/23%)
Total number of individuals examined in systematic review and meta-analysis(n = 13)/all sample	Paramedicine students (1064/1623)
Age (Mean) (n = 11)	24.7 years; (range: 17–53 years)
Paramedic students (n = 13) men / women/prefer not to say	(698/56.5%/ 533/43.2%/ 3/ > 1%

PTSD, anxiety and depression studies included in the meta-analysis were examined using comprehensive meta-analysis (CMA) software. A random-effects meta-analysis was conducted using pooled mean prevalence estimates and expressed as an event rate. The results were calculated using a 95% confidence interval (CI) and *p*-value. A high heterogeneity was expected, given the probable levels of heterogeneity. The random effect is preferred in such cases; however, the fixed model is favoured for prevalence studies to maintain the weight of studies. Therefore, both the random and fixed effects were displayed in the results (M. [12, 35]). Where appropriate, heterogeneity factors were assessed using the Higgins inconsistency test (I^2^) and *p*-value. A *p-value* below 0.05 was considered statistically significant [25]. The risk of publication bias was examined through Egger’s test and Begg’s funnel plot, which were prepared using CMA software [9, 15]. See Appendix 1, 2, and 3C. No subgroup analyses were performed due to insufficient data available for analysis.

## Results

### Study characteristics

**Fig. 1:**
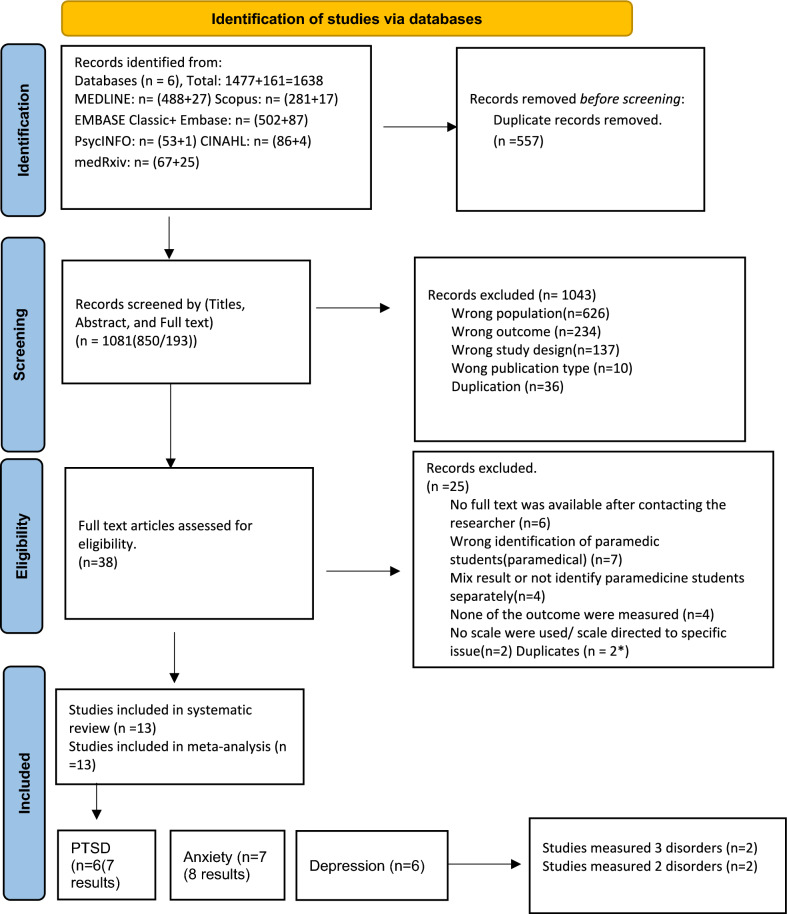
“PRISMA 2020 Flow Diagram” [42]

A total of 1638 articles from six databases were identified (See Fig. 1). After removing duplicates, 1,081 studies remained. During the title and abstract screening phase, 850 studies were excluded as they did not match the current systematic review criteria; 193 studies were screened fully. Only 38 studies met the inclusion criteria, and a further 23 of these were excluded as the researchers could not obtain the relevant data, even after contacting the study authors. Thus, 13 studies were included in the systematic review [6, 8, 13, 16, 21, 22, 32, 34, 37–39, 51, 55, 56], and met the criteria for meta-analysis. Of these 13 studies, six were included in the final set for PTSD, seven for anxiety, and six for depression. Please refer to Fig. 1 for the PRISMA diagram.

Articles were primarily excluded in the first and second screening stages due to the study population (n = 626), outcomes (n = 234), design (n = 137), and publication type (n = 10). During the eligibility stage, articles were primarily excluded because the rate of each disorder of interest among paramedic students could not be identified, a lack of detailed results provided for each scale, and reporting of the mean results only.

A total of 1623 participants were included in the review; from this sample, 1064 paramedic students from 13 studies conducted across 10 countries were included in the meta-analyses. The included studies were published between 1996 and 2022, with the number of studies increasing with time.

Six studies were cross-sectional, including a prevalence-based study; two were mixed methods; two were longitudinal, including a study that provided outcomes from two different collection points as well as baseline prevalence data for the second dataset; three were cohort studies; and two used alternative study designs. Regarding the sampling techniques, 12 studies used non-random methods (purposeful sampling and convenience sampling), while the remaining three used random or census methods. If the sample methods were not mentioned, they were listed as non-random methods.

Regarding gender, 56% of the participants were male and 43% were female. In two studies, no gender information was listed. All the studies used self-report scales to identify the prevalence of mental health conditions, and no study used a clinical diagnostic interview. For further information, see Appendix 2, 3, 4B.

## Mental health outcomes

### Prevalence of posttraumatic stress disorder

Six studies reported symptoms of PTSD among paramedic students. These symptoms were assessed using the following four scales: the PTSD Checklist for DSM-5 (PCL-5), the Posttraumatic Stress Diagnostic Scale (PDS), the Davidson Trauma Scale (DTS), and Keane’s MMPI scale (PK) for PTSD. The pooled prevalence estimate of moderate PTSD was 17.9% (95% CI, 14.8–21.6%; see Appendix Table E), with a range of 5–22%. The heterogeneity was low (I2 = 1%, p < 0.001), reflecting variance in true effects rather than sampling error. This was evidenced through the Q-value, which was 6.055 with six degrees of freedom and p 0.417. The pooled estimate of mild PTSD was not presented due to a lack of available data for four studies.

#### Prevalence of anxiety

Seven studies reported symptoms of anxiety among paramedic students. Five scales were used in these studies, including the seven-item Generalised Anxiety Disorder Scale (GAD-7), Depression Anxiety Stress Scales (DASS-21), Westside Test Anxiety Scale (WTA), Trait Anxiety Inventory (TAI), and State-Trait Anxiety Inventory (STAI). The pooled estimated prevalence of moderate anxiety was found to be 56.4% (95% CI 35.9%, 75%). While the prevalence of mild anxiety, according to the analysis of four studies, was estimated at 27.1% (95% CI 15.8%, 42.4%). The estimated range of moderate anxiety on each study varied between 24.3 and 94%. The statistical analysis revealed high heterogeneity (I^2^ = 96%, *p* < 0.001) among the studies, indicating a difference in true effects rather than sampling error. The Q-value was 155.907 with seven degrees of freedom and *p* < 0.001. Subgroup analyses were not conducted due to insufficient studies for comparison.

#### Prevalence of depression

Six studies reported symptoms of depression among paramedic students. These studies used six scales: the Beck Depression Inventory and Revised Beck Depression Inventory (BDI & BDI-II), the DASS-21, the Center for Epidemiological Studies Depression Scale (CES-D), the nine-item Patient Health Questionnaire (PHQ-9), and the Kessler Psychological Distress Scale (K10). Based on six scales, the prevalence of moderate depression was estimated at 34.7% (95% CI 0.234–0.481). The range of prevalence estimates across the studies was between 8 and 56.2%. However, there was a high level of heterogeneity between the studies, with an I^2^ of 83%, indicating a difference in true effects rather than sampling error. The Q-value is 28.616 with 5 degrees of freedom and *p* < 0.001. Unfortunately, the pooled estimate of mild depression could not be presented due to the lack of available data. Further, subgroup analyses were not conducted as there was insufficient data to make comparisons.

### Publication bias

Various methods were used to evaluate publication bias. For PTSD, Begg’s funnel plot indicated symmetry (see graph 1c), while Egger’s test showed non-significance with an intercept (B0) of  – 1.685 and a 95% confidence interval ( – 3.99623, 0.62586) and a *p*-value of 0.0598. The null hypothesis was not rejected with a criterion alpha of 0.100, as the actual effect size varied among the studies. The prediction interval was estimated to be between 13.8 and 23%, with the true effect size falling within this range for 95% of similar populations. For anxiety, Begg’s funnel plot suggested publication bias (see graph 2c). Still, Egger’s test did not provide any significant evidence, with an intercept (B0) of 5.71895, a 95% confidence interval ( – 3.22881, 14.66671), and a p-value of 0.08443. The null hypothesis was rejected with a criterion alpha of 0.100, as the true effect size differed in all studies. The prediction interval was estimated to be between 6.3 and 96.1%, with the true effect size in 95% of all comparable populations falling in this range. For depression, while there was possible publication bias due to a slightly asymmetric Begg’s funnel plot, Egger’s test (*p* = 0.18) found no significant evidence of bias, with an intercept (B0) of  – 2.19777, a 95% confidence interval (-8.28963, 3.89409), and a *p*-value of 0.18659. The null hypothesis was rejected with a criterion alpha of 0.100, as the true effect size differed in all studies. The prediction interval was estimated to be between 8.2 and 75.9%, with the true effect size in 95% of all comparable populations falling within this range.

### Sensitivity analyses

In order to test sensitivity, the leave-one-out method was used, as described by Higgins et al. [61]. The prevalence of PTSD remained unchanged as a result of the application, with minor changes ranging from 0.06% to 13.5% in six studies and remaining significant. This suggests that a single study did not influence the findings, as two studies showed an increase of 2.3% and a decrease of 5.3%, respectively. For anxiety, the prevalence changed in three studies, while it increased from 5.4% to 10% in four studies. The remaining three studies showed an increase of 3.9–5.6%, although the relative weight remained almost the same in five studies. Regarding depression, the prevalence remained unchanged as a result of the application, with slight changes ranging from 0.07% to 3% in four studies. However, in only two studies, the difference was between 3 and 7%, indicating that two to four studies influenced the results for anxiety and depression.

## Discussion

To our knowledge, this is the first systematic review and meta-analysis examining the prevalence of anxiety, depression, and post-traumatic stress disorder among the paramedic student population. Accordingly, the current project presents a major contribution to the literature as it illustrates the prevalence of mental health conditions using 15 studies from 10 countries examining a total of 1,392 paramedic students. The prevalence demonstrated high rates of moderate-to-high anxiety (56%), depression (34%), and PTSD (17.9%). These findings are consistent with a systematic review of paramedics, which reported lower rates of PTSD than other mental health conditions [43].

The pooled estimate for mental health disorders among paramedicine students was higher than those found in similar reviews of qualified paramedics [17, 28, 41, 43]. It is possible that this is due to stressors related to the experiences involved in paramedicine training programmes. The clinical training for paramedicine varies by country and university,some students are sent to prehospital providers and different hospital departments, while others receive further training facilities. This diversity of experiences adds to the complexity of their training and the challenges they face. Furthermore, some programs send students for clinical training as early as their first month of the program. The training focuses on monitoring the field and caring for patients in time-sensitive situations, in a limited space, and with several cases and challenges encountered. It is also worth noting that the current prevalence of mental health conditions among paramedic students is higher than in other student populations, indicating the need for collective attention and action to prevent adverse effects on paramedicine student wellbeing [7, 23, 44] and educational outcomes [31].

The results of our systematic review have shown that paramedic students are more likely to experience anxiety than other mental health conditions. This is consistent with wider trends in mental health disorder occurrence, which highlight anxiety as the most common mental health disorder [18]. This could also be attributed to the COVID-19 pandemic, which was initially associated with an increase in anxiety worldwide [56]. However, more recent meta-analyses comparing mental health symptoms before and after the COVID-19 pandemic suggests any initial differences in mental health symptoms in the general population have since reduced to pre-pandemic levels, with only a slight maintained increase in healthcare providers [45, 49, 50].

The findings revealed substantial heterogeneity among all the mental health outcomes. It was particularly important to consider the high heterogeneity between anxiety, depression, and PTSD when interpreting the estimated pooled prevalence in our meta-analysis of the percentage of variability (I^2^). Each disorder had between six and six to seven studies with eight intakes, such as PTSD. Generally, estimates of heterogeneity based on fewer than 10 studies are unreliable [11]. As a result of the limited data available, a subgroup analysis could not be performed to test for evidence related to content or screening tools. Furthermore, we could not fully explain the high heterogeneity, particularly given the limited number of studies and the different scales used.

Regarding PTSD, studies that used the PCL-5 showed a similar prevalence rate (i.e., between 16 and 17%). However, the sample size and the date of the study showed no significance. Regarding depression, no scales showed any similar patterns to anxiety and PTSD. The studies published since the COVID-19 pandemic reported higher rates of depression than those published before, but it should be noted that all included studies were conducted during earlier phases of the pandemic and wider trends suggest that rates of depression have since returned to pre-pandemic levels [45, 49, 50].

### Strengths and limitations

The primary strength of this systematic review is that it focuses on an international population with no limits to specific geographic areas or academic systems. Further, the study was registered on Prospero and followed the PRISMA guidelines to ensure methodological rigour. Two reviewers screened all the studies in the title and abstract stages, with a third reviewer conducting a full review for the data extraction.

However, there are several limitations to the employed methodology. The majority of the studies in the review used non-random sampling methods, which could generate selection bias. Some studies may have been missed as only articles written in English were included in the systematic review. Additionally, while preprint study was included to gather as much data as possible, the results of such studies could change from preprint to publication but that was not the case in the studies included.

The findings revealed substantial heterogeneity among all the mental health outcomes. It was particularly important to consider the high heterogeneity between anxiety, depression, and PTSD when interpreting the estimated pooled prevalence in our meta-analysis of the percentage of variability (I^2^). Each disorder had between six to seven studies with eight intakes, such as anxiety. Generally, estimates of heterogeneity based on fewer than 10 studies are unreliable [11]. As a result of the limited number of studies available to be included in the review, it was not possible to conduct subgroup analyses or meta-regression to investigate moderating effects or to compare for differences according to factors such as screening tools used [64, 65]. Furthermore, we could not fully explain the high heterogeneity, particularly given the limited number of studies and the different scales used. This issue was particularly evident in studies from countries with unconventional paramedicine training systems, such as India and Iran. Further, although the researchers attempted to contact study authors who did not list their full outcomes, many did not respond, despite being contacted over three times in a six-month period.

Further, the samples used in the studies were limited, with some being from one setting and one university only. Thus, the approach lacked randomisation. Begg’s funnel plots revealed signs of slight-to-high publication bias, but Egger’s test results did not reflect the same bias. The limited sample and lack of available data undoubtedly contributed to these discrepancies.

## Conclusion

The present systematic review and meta-analyses provide the most comprehensive information on the prevalence of anxiety, depression, and PTSD among international paramedic students to date. Results suggest that paramedic students are at risk for common mental health conditions, particularly anxiety. This review can guide future research on the mental health of paramedic students internationally, a population that faces numerous and varied challenges and stressors with long-term negative effects. All parties involved, from academic administrators to service providers, need to take decisive action to meet the needs and address the concerns of paramedic students before they enter the field. Globally, universities must implement more support initiatives and improve existing mental health interventions in paramedicine programmes, particularly in collaboration with paramedic students.

## Electronic supplementary material

Below is the link to the electronic supplementary material.

Supplementary file1 (DOCX 41 KB)

## Data Availability

The datasets generated and/or analysed during the current study are publicly available in published studies.

## Appendix: 1F Data extraction PTSD

**Table:**

SN	Study/Year	Country	Scale_a_	Research design	Sample size all/Paramedic students	Sampling method	Gender/mean Age	Prevalence rate (%)	Mean	SD	SE	Effect size weighted average prevalence (%)	CI	Q between subgroup tests p value
1	[16]	South Africa	Davidson trauma scale (DTS)	Cross-sectional	130	Non random	M.84(63.6) F.48(36.4) /22	16%	1.18	N/A	0.11	Unavailable	95% 0.93–1.43	N/A
2	McKinnon et al [37]	United Kingdom	Posttraumatic Stress Disorder Checklist for DSM-5 (PCL-5)	Mix method longitudinal	89	Opportunistic sampling method	M.33(38.2%) F.55(61.8%) /26	16.8%(N = 15)–20.2%(N = 18)	B15.52/ F15.39	B16.1/ F15.8	B. 0.08	0.45***	[0.48–0.81[	*p* < 0.001
3	[34]	Australia	Posttraumatic Stress Diagnostic Scale (PDS)	Cross-sectional	42	Non random	M.35 F. 7/ 35	5% 17% mild	3.81	4.41	(0.08)	Unavailable	Unavailable	N/A
4	Mehta et al. 2021	Australia	Posttraumatic Stress Disorder Checklist for DSM-5 (PCL-5)	longitudinal	80	Non random	M.15(38.5) F.48(61.5) /23	N/A	B16.82/ F12.82	N/A	2.28/2.27	Unavailable	Unavailable	N/A
5	[39]	Australia	1- The Posttraumatic Stress Disorder Checklist for DSM-5(PCL-5) 2- The Posttraumatic Growth Inventory X	Pilot study	47	Non random	M.28 (59.6) F.18 (38.3) Intersex 1 (2.1)/ 23	1. 17.02%	1–16.81 2- 72.4	n/a	1- 2.14 2- 4.28	Unavailable	Unavailable	N/A
6	[22]	Canada	Davidson trauma scale(DTS)	Cohort	13(6 done PTSD)	Convenience sample	M.8(38.5) F.5(61.5)/25	16.6%	7.77	11.35	N/A	Unavailable	unavailable	n/a
7	[21]	USA	The Posttraumatic Stress Disorder (PK) scale	Cohort/ Dissertation	225/105	Convenience sample	M.93 F.12/27	21.9%	N/A	N/A	N/A	Unavailable	Unavailable	ps < 0.0001

*PCL* PTSD Checklist. *PTDS* Posttraumatic Stress Diagnostic Scale. *PDS* Posttraumatic Stress Diagnostic Scale. *N* Number of participant See the study coding in Appendix 1

^a^Scale of Posttraumatic Stress Symptoms. *DTS* Davidson Trauma Scale

## Appendix: 2F Data extraction anxiety

**Table:**

SN	Study/Year	Country	Research design	Scale_a_	Sample size all/Paramedic students	Sampling method	Gender/Mean Age	Prevalence rate (%)	Mean	SD	SE	Effect size weighted average prevalence (%)	CI	Q between subgroup tests p value
1	McKinnon et al. [37]	UK	Mix methods Longitude	GAD7	89	Opportunistic sampling method	M.33(38.2%) F.55(61.8%) /26.36	Mild:30% 27%	B.6.25 f.6.12	5.23 5.2	0	n/a	Unavailable	N/A
2	Williams et al. [13]	AU	Mix methods	GAD7	151	Convenience sample	M: 36 (23.8) F: 113 (74.8) Prefer NTS: 2 (1.3) /n/a	35% mild 27%	7.67	4.85	Unavailable	Unavailable	Between gender 95% CI: (1.02, 4.12)	Between gender p = 0.045
3	[56]	New Zealand	Mix methods	WTA	117	All students/ census method	M.43 F. 82 gender diverse. 1/ 20–29	70%	33.76	± 7.23	Unavailable	N/A	Unavailable	N/A
4	[22]	Canada	Cohort	STAI & TAI	13	Convenience sample	M.8(38.5) F.5(61.5)/25	13/16 = 81% 15/16 = 94% Moderate and higher Sever 19% 31%	44.77 49.54	8.08 7.29	Unavailable	N/A	Unavailable	N/A
5	[6]	KSA	Cross-sectional	GAD7	181	All students/ census method	M. 133 (79.2%) F. 48 (75%) /22	21%m 33%f Both = 24.3%			Unavailable	N/A	Unavailable	N/A
6	[51]	Turkey	Cross-sectional	TAI	185/108	Random sampling	M. 108 (100%) F. 0(0%)/ 20y/o	37.1%	34.02	5.45	N/A	N/A	N/A	0.07
7	[32]	Tunisia	Cross-sectional	DASS21	366/114	Convenience sample	M. 22 (6%) F. 344 (94%) / n/a	91.2%			N/A	N/A	N/A	N/A

*GAD-7* 7-item Generalised Anxiety Disorder Scale, *DASS* Depression Anxiety Stress Scales, *WTA* Westside Test Anxiety Scale, *TAI* Trait Anxiety Inventory, *STAI* state-trait anxiety inventory

## Appendix: 3F Data extraction depression

**Table:**

SN	Study/Year	Country	Research design	Scale	Sample size all/Paramedic students	Sampling method	Gender/Mean Age	Prevalence rate (%)	Mean	SD	SE	Effect size weighted average prevalence (%)	CI	Q between subgroup tests p value
1	[16]	South Africa	Cross-sectional	CES-D	130	Non random	M.84(63.6) F.48(36.4) /22	28%	1.245 Odds ration.1.23	Unavailable	0.07	Unavailable	1.07–1.42	N/A
2	McKinnon etal. 2021	UK	Mix methods Longitudinal	PHQ-9	89	Opportunistic sampling method	M.33(38.2%) F.55(61.8%) /26	30%(32% mild)	B. 6.73 F.6.39	B. 5.76 F.5.23	Unavailable	Unavailable	N/A	N/A
3	Mehta et al. 2021	AU	Longitudinal	K10(for distress)	78 39 b + f	Non random	M.15(38.5) F.48(61.5) /23	43.6%	B.18.77 F.18.69	Unavailable	1.1 1.05	Unavailable	N/A	N/A
4	[8]	Iran	Descriptive-correlative study	(BDI-II)	119/9	Convenient sampling	n/a	22%		Unavailable`	Unavailable	Unavailable	N/A	N/A
5	[22]	Canada	Cohort	BDI	41/13	Convenience sample	M.8(38.5) F.5(61.5)/25	8%	4.46	3.4	Unavailable	Unavailable	N/A	N/A
6	[32]	Tunisia	Cross-sectional	DASS21	366/114	Convenience sample	M. 22 (6%) F. 344 (94%)/ n/a	56.2%	N/A	N/A	N/A	N/A	N/A	N/A

*aBDI* Beck Depression Inventory, *BDI-II* Beck Depression Inventory, *DASS* Depression Anxiety Stress Scales, *CES-D* Centre for Epidemiological Studies Depression Scale, *PHQ-9* The Nine items Patient Health Questionnaire, *K10* The Kessler Psychological Distress Scale
