# Supplementary material for: Prevalence of anxiety, depression, and post-traumatic stress disorder among paramedic students: a systematic review and meta-analysis
Source: Soc Psychiatry Psychiatr Epidemiol. 2024 Sep 12;60(3):563–78. doi: 10.1007/s00127-024-02755-6 (PMC11870987; doi:10.1007/s00127-024-02755-6)
Supplement: Supplementary file 1 — Supplementary file1 (DOCX 41 KB) [file 127_2024_2755_MOESM1_ESM.docx]

**Supplemental material**

| Table 1. Completed PRISMA checklist. | 2 |
| --- | --- |
| Searching methods 2. Search strategy example for *MEDLINE(R)* | 4-8 |

| **Section/topic** | **#** | **Checklist item** | **Reported on page #** |
| --- | --- | --- | --- |
| **TITLE** | | |  |
| Title | 1 | Identify the report as a systematic review, meta-analysis, or both. | 2 |
| **ABSTRACT** | | |  |
| Structured summary | 2 | Provide a structured summary including, as applicable: background; objectives; data sources; study eligibility criteria, participants, and interventions; study appraisal and synthesis methods; results; limitations; conclusions and implications of key findings; systematic review registration number. | 3 |
| **INTRODUCTION** | | |  |
| Rationale | 3 | Describe the rationale for the review in the context of what is already known. | 4 |
| Objectives | 4 | Provide an explicit statement of questions being addressed with reference to participants, interventions, comparisons, outcomes, and study design (PICOS). | 5 |
| **METHODS** | | |  |
| Protocol and registration | 5 | Indicate if a review protocol exists, if and where it can be accessed (e.g., Web address), and, if available, provide registration information including registration number. | 5 |
| Eligibility criteria | 6 | Specify study characteristics (e.g., PICOS, length of follow-up) and report characteristics (e.g., years considered, language, publication status) used as criteria for eligibility, giving rationale. | 7 |
| Information sources | 7 | Describe all information sources (e.g., databases with dates of coverage, contact with study authors to identify additional studies) in the search and date last searched. | 5&8 |
| Search | 8 | Present full electronic search strategy for at least one database, including any limits used, such that it could be repeated. | 5 |
| Study selection | 9 | State the process for selecting studies (i.e., screening, eligibility, included in systematic review, and, if applicable, included in the meta-analysis). | 7 |
| Data collection process | 10 | Describe method of data extraction from reports (e.g., piloted forms, independently, in duplicate) and any processes for obtaining and confirming data from investigators. | 8 |
| Data items | 11 | List and define all variables for which data were sought (e.g., PICOS, funding sources) and any assumptions and simplifications made. | 9 |
| Risk of bias in individual studies | 12 | Describe methods used for assessing risk of bias of individual studies (including specification of whether this was done at the study or outcome level), and how this information is to be used in any data synthesis. | 8&9 |
| Summary measures | 13 | State the principal summary measures (e.g., risk ratio, difference in means). | 8&9 |
| Synthesis of results | 14 | Describe the methods of handling data and combining results of studies, if done, including measures of consistency (e.g., I^2^) for each meta-analysis. | 9 |

| **Section/topic** | **#** | **Checklist item** | **Reported on page #** |
| --- | --- | --- | --- |
| Risk of bias across studies | 15 | Specify any assessment of risk of bias that may affect the cumulative evidence (e.g., publication bias, selective reporting within studies). | 9 |
| Additional analyses | 16 | Describe methods of additional analyses (e.g., sensitivity or subgroup analyses, meta-regression), if done, indicating which were pre-specified. | 8 |
| **RESULTS** | | |  |
| Study selection | 17 | Give numbers of studies screened, assessed for eligibility, and included in the review, with reasons for exclusions at each stage, ideally with a flow diagram. | 9-10 |
| Study characteristics | 18 | For each study, present characteristics for which data were extracted (e.g., study size, PICOS, follow-up period) and provide the citations. | 9-10 |
| Risk of bias within studies | 19 | Present data on risk of bias of each study and, if available, any outcome level assessment (see item 12). | 13 |
| Results of individual studies | 20 | For all outcomes considered (benefits or harms), present, for each study: (a) simple summary data for each intervention group (b) effect estimates and confidence intervals, ideally with a forest plot. | 11-13 |
| Synthesis of results | 21 | Present results of each meta-analysis done, including confidence intervals and measures of consistency. | 11-13 |
| Risk of bias across studies | 22 | Present results of any assessment of risk of bias across studies (see Item 15). | 13 |
| Additional analysis | 23 | Give results of additional analyses, if done (e.g., sensitivity or subgroup analyses, meta-regression [see Item 16]). | 13-14 |
| **DISCUSSION** | | |  |
| Summary of evidence | 24 | Summarize the main findings including the strength of evidence for each main outcome; consider their relevance to key groups (e.g., healthcare providers, users, and policy makers). | 14-16 |
| Limitations | 25 | Discuss limitations at study and outcome level (e.g., risk of bias), and at review-level (e.g., incomplete retrieval of identified research, reporting bias). | 16-17 |
| Conclusions | 26 | Provide a general interpretation of the results in the context of other evidence, and implications for future research. | 17 |
| **FUNDING** | | |  |
| Funding | 27 | Describe sources of funding for the systematic review and other support (e.g., supply of data); role of funders for the systematic review. | 18 |

**Methods 2. Search strategy**

| **Concepts** | 1. **MEDLINE** | | 1. **EMBASE** | | 1. **CINAHL** | | 1. **Scopus** | |
| --- | --- | --- | --- | --- | --- | --- | --- | --- |
|  | MeSH terms | Keywords | EMTREE terms | Keywords | MH Terms | keywords | Topic Terms | Keywords |
| **Concept 1**  Mental  health | 1. mental health/ 2. exp mental disorders / 3. Anxiety/ 4. Psychological Trauma 5. Panic/ 6. exp Stress, Psychological/ 7. Depression/ 8. exp posttraumatic or "post traumatic" or PTSD | 1. mental health 2. mental disorders 3. anxiety 4. depression 5. post traumatic stress disorder 6. stress 7. wellbeing | 1. exp mental health/ 2. exp mental disorders / 3. Anxiety/ 4. Psychological Trauma 5. Panic/ 6. exp Stress, Psychological/ 7. Depression/ 8. exp posttraumatic stress disorder/ 9. Psychological Distress/ 10. exp Adaptation, Psychological/ | 1. mental health 2. mental disorders 3. anxiety 4. depression 5. post traumatic stress disorder 6. stress 7. wellbeing | 1. ((MH "Mental Health") 2. (MH "Mental Disorders+") 3. (MH "Anxiety+") 4. (MH "Cumulative Trauma Disorders") 5. stress disorders, post-traumatic or ptsd 6. (MH "Depression+") 7. (MH "Adaptation, Psychological") 8. (MH "Emotions") | 1. Mental Health 2. Mental Disorders 3. Anxiety 4. Cumulative Trauma Disorders 5. stress disorders, post-traumatic or ptsd 6. Depression 7. Adaptation, Psychological 8. Emotions | 1. KEY ( "mental health" ) 2. KEY ( "Anxiety" ) 3. KEY ( "Stress Disorders, Post-Traumatic" ) 4. KEY ( "psychotrauma" ) 5. KEY ( "Depression" ) 6. KEY ( "stress disorders, traumatic, acute" ) 7. KEY ( "coping" ) 8. KEY ( " Adaptation, Psychological" ) | 1. mental health 2. Anxiety 3. Psychological Trauma 4. Panic 5. Stress Disorders, Post-Traumat 6. Depression 7. coping 8. Adaptation, Psychological |

| **Concept 2**  Paramedic  Student | 1. exp Students, Health Occupations/ 2. exp paramed* or emergency medical technician*/ 3. exp Emergency Medical Technicians/ and students/ 4. Student* or undergrad* or trainee* or intern*).ti,ab. | 1. Paramedic* students 2. Undergrade 3. trainee* 4. intern 5. EMT 6. Paramedic* 7. Emergency Medical Service* 8. Emergency Medical Technician* | 1. exp Health student/ or paramedical student/ 2. exp Emergency Medical Technicians/ and students/ 3. exp paramed*/ 4. emergency medical technician*/ 5. (student* undergrad* trainee* intern*) | 1. Health student 2. paramedical student 3. Emergency Medical Technicians 4. Students 5. Undergrade. | 1. MH "Emergency Medical Technicians") 2. Students 3. paramed* or emergency medical technician*) 4. student* or undergrad* or trainee* or intern*)) | 1. student 2. paramedical student 3. Emergency Medical Technicians 4. Undergrade. | 1. KEY ( emergency  AND medical  AND technicians  AND  students ) 2. KEY ( "Students"  W/3  health  AND occupations/ ) 3. TITLE-ABS-KEY ( ( "paramed*" )  W/3  ( student*  OR  undergrad* ) ) | 1. emergency medical technicians...D students 2. Students health occupations 3. paramed student undergrad |
| --- | --- | --- | --- | --- | --- | --- | --- | --- |

| **Concepts** | 1. **PsycINFO** | |
| --- | --- | --- |
|  | MeSH descriptor | Keywords |
| **Concept 1**  Mental  health | 1. exp Mental Health/ 2. exp Anxiety/ 3. exp Posttraumatic Stress Disorder/ 4. exp Panic/ 5. exp Adjustment Disorders/ 6. exp Acute Stress Disorder/ 7. stress/ 8. exp "Resilience (Psychological)"/ | 1. Mental Health 2. Anxiety 3. Posttraumatic Stress Disorder 4. depression 5. Panic 6. Adjustment Disorders 7. Acute Stress Disorder 8. stress 9. Resilience 10. Psychological |
| **Concept 2**  Paramedic  Student | 1. exp Allied Health/ 2. exp Emergency Services/ 3. exp Emergency Personnel/ 4. exp Paramedics/) and students/ 5. paramed* 6. emergency medical technician* 7. student* or undergrad* or trainee* or intern*. | 1. Allied Health 2. Emergency Personnel 3. students 4. paramedic 5. emergency medical technician |

**Methods 2. Search strategy example for *MEDLINE(R)***

1. Database: ***Ovid MEDLINE(R) ALL <1946 to February 10, 2023>***

| 1. *Ovid MEDLINE(R) ALL <1946 to February 10, 2023>* | | |
| --- | --- | --- |
| # | **Query** | **Results** |
| 1 | Students, Health Occupations/ | 3321 |
| 2 | Emergency Medical Technicians/ and students/ | 33 |
| 3 | ((paramed* or emergency medical technician*) adj5 (student* or undergrad* or trainee* or intern*)).ti,ab. | 610 |
| 4 | or/1-3 [paramedical students] | 3907 |
| 5 | mental health/ | 58375 |
| 6 | mental health.ti,ab. | 199479 |
| 7 | exp mental disorders/ | 1411857 |
| 8 | mental disorder*.ti,ab. | 47419 |
| 9 | Anxiety/ | 103623 |
| 10 | Anxiety Disorders/ | 40451 |
| 11 | Psychological Trauma/ | 1843 |
| 12 | "trauma and stressor related disorders"/ | 120 |
| 13 | Panic/ | 2689 |
| 14 | Panic Disorder/ | 7252 |
| 15 | Stress Disorders, Post-Traumatic/ | 40288 |
| 16 | (anxiety or trauma or panic or posttraumatic or "post traumatic" or PTSD).ti,ab. | 563816 |
| 17 | exp Stress, Psychological/ | 150927 |
| 18 | psychotrauma.ti,ab. | 110 |
| 19 | Depression/ | 147128 |
| 20 | Adjustment Disorders/ | 4305 |
| 21 | stress disorders, traumatic, acute/ | 536 |
| 22 | stress disorders, traumatic/ | 750 |
| 23 | Psychological Distress/ | 3840 |
| 24 | Resilience, Psychological/ | 8168 |
| 25 | coping.ti,ab,kw. | 68958 |
| 26 | exp Adaptation, Psychological/ | 138532 |
| 27 | (anxiety or trauma or panic or posttraumatic or "post traumatic" or PTSD or stress or psychotrauma or depress* or neuros*s or "adjustment disorder*" or distress or resilience or wellbeing or "well being").ti,ab. | 2002297 |
| 28 | or/5-27 [mental health] | 3310032 |
| 29 | 4 and 28 [paramedical students and mental health] | 514 |
| 30 | limit 29 to yr="2022 -Current" | 27 |
